# Vascular age vs. chronological age in operative risk stratification

**DOI:** 10.3389/fcvm.2026.1780214

**Published:** 2026-05-14

**Authors:** Kenyon Sprankle, Christopher Potestio, Vonna Jonna Venkatasai S., Meghna Matthews, Lawrence J. Mulligan

**Affiliations:** 1 Cooper Medical School of Rowan University, Camden, NJ, United States; 2 Department of Anesthesiology, Cooper University Hospital, Camden, NJ, United States

**Keywords:** arterial stiffness, coronary artery disease, early vascular aging, heart failure, hypertension

## Abstract

Early vascular aging (EVA) describes a disconnect between an individual's chronological age and their biological vascular age. It is thought to be caused by endothelial dysfunction, oxidative stresses, and chronic low-grade inflammation. Evidence collected over the past three decades suggests that vascular aging may be linked to and exacerbated by conditions such as hypertension (HTN), coronary artery disease (CAD) and heart failure with preserved ejection fraction (HFpEF). This accelerated vascular aging results in younger individuals having vascular profiles and perioperative risks that would be expected in individuals of a more advanced chronological age.

## Introduction

**Figure 1:**
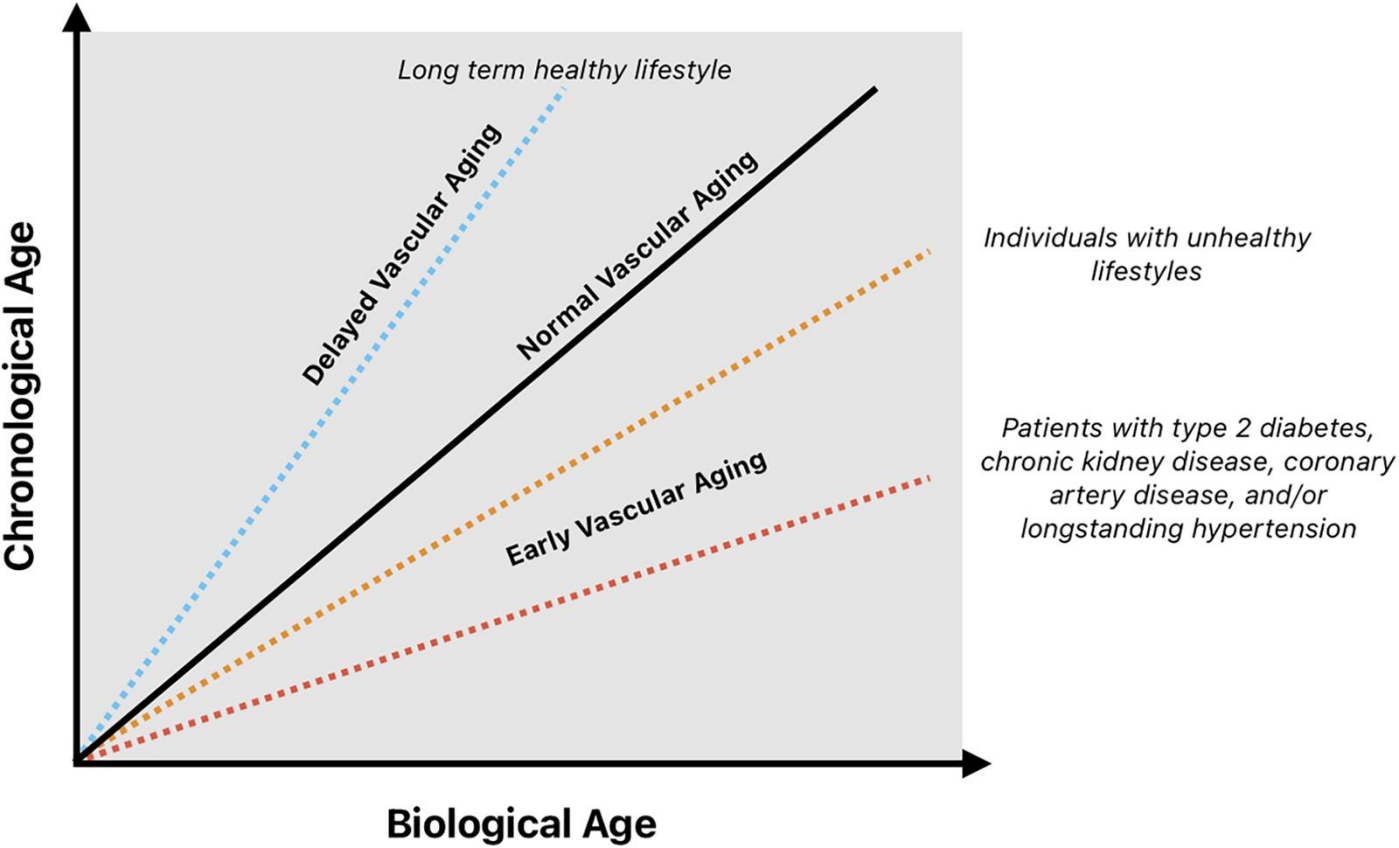
Separation of chronological and biological age in different individuals: this schematic demonstrates how individuals may follow trajectories of delayed, normal, or premature vascular aging depending on genetic factors, lifestyle, comorbid disease, and environmental exposures. The divergence between biological and chronological age provides a framework for understanding Early Vascular Aging (EVA) and its clinical implications.

Chronological age has been a useful proxy for biological/vascular aging. However, in medical literature, there is growing recognition of vascular age as a distinct entity from chronologic age. Early vascular aging (EVA) is a phenomenon of vascular stiffening, endovascular dysfunction and lumen diameter shifts that can progress more rapidly than one's chronologic age would suggest (1). The mismatch between pathologic EVA and chronological age may help explain why younger patients with comorbidities such as hypertension, coronary disease, or heart failure sometimes manifest complications (e.g., end-organ injury, delayed postoperative wound healing) that are more commonly seen in older individuals (see Figure 1).

At the core of their pathophysiologies hypertension (HTN), coronary artery disease (CAD), and heart failure with preserved ejection fraction (HFpEF) can advance an individual's vascular age significantly (see Figure 1). Understanding how these comorbid processes impact an individual over the course of their lives has important implications inside and outside the operating room.

In this manuscript we aim to investigate how HTN, CAD and HFpEF serve as accelerators and potential consequences of vascular aging and how we may be able to integrate vascular age into perioperative decision support and patient risk stratification.

## Quantification of a patient's vascular age through perioperative assessment

Early vascular aging is often discussed qualitatively however multiple large epidemiologic studies have demonstrated that vascular age can be partially determined quantitatively by analyzing cardiovascular risk markers. In the Framingham Heart Study, we see that cardiovascular risk factors such as HTN, glucose intolerance and echocardiographic evidence of left ventricular hypertrophy are closely associated with decreased survival as well as increased morbidity independent of chronological age (2). The combination of a patient's cardiovascular risk factors predicted survival more accurately than chronological age alone. This helps support the concept of a vascular age that is separate and distinct from the chronological age (3, 4).

Pulse wave velocity (PWV), is a noninvasive measure of a patient's vascular stiffness is the most reliable predictor of cardiovascular and all-cause mortality across multiple patient populations (3, 5). A large systematic review and meta-analysis showed that a higher PWV is independently associated with increased mortality risk even when you consider other conventional risk factors (3). This further integrates PWV as a marker of vascular aging and helps cement its usefulness in determining a patients vascular age (5).

Population studies demonstrate that in otherwise healthy adults, carotid-femoral pulse wave velocity (cfPWV) increases predictably with age. In those with cfPWV exceeding commonly accepted thresholds (>10 m/s) their arterial profiles may better mirror individuals who are one to two decades older (1, 5–7). Patients who have well-controlled HTN and those with long standing/poorly controlled HTN see shifts in vascular ages of five to ten years and ten to fifteen years respectively (4, 8, 9). CAD and its underlying pathophysiology can produce vascular phenotypes comparable to individuals ten to twenty years older than what would be expected for their chronologic age (2, 10, 11). HFpEF is associated with significantly elevated arterial stiffness compared with age-matched controls which reflects advanced ventricular-arterial uncoupling as well as vascular aging on the order of one to two decades (12–14). These cumulative findings support a semi-quantitative basis of understanding an individual's vascular age beyond their chronologic age alone when considering their comorbidities. It should be noted that many of these conditions do not occur in isolation and overlap may make these estimates less reliable.

## Literature review

### Hypertension

**Figure 2:**
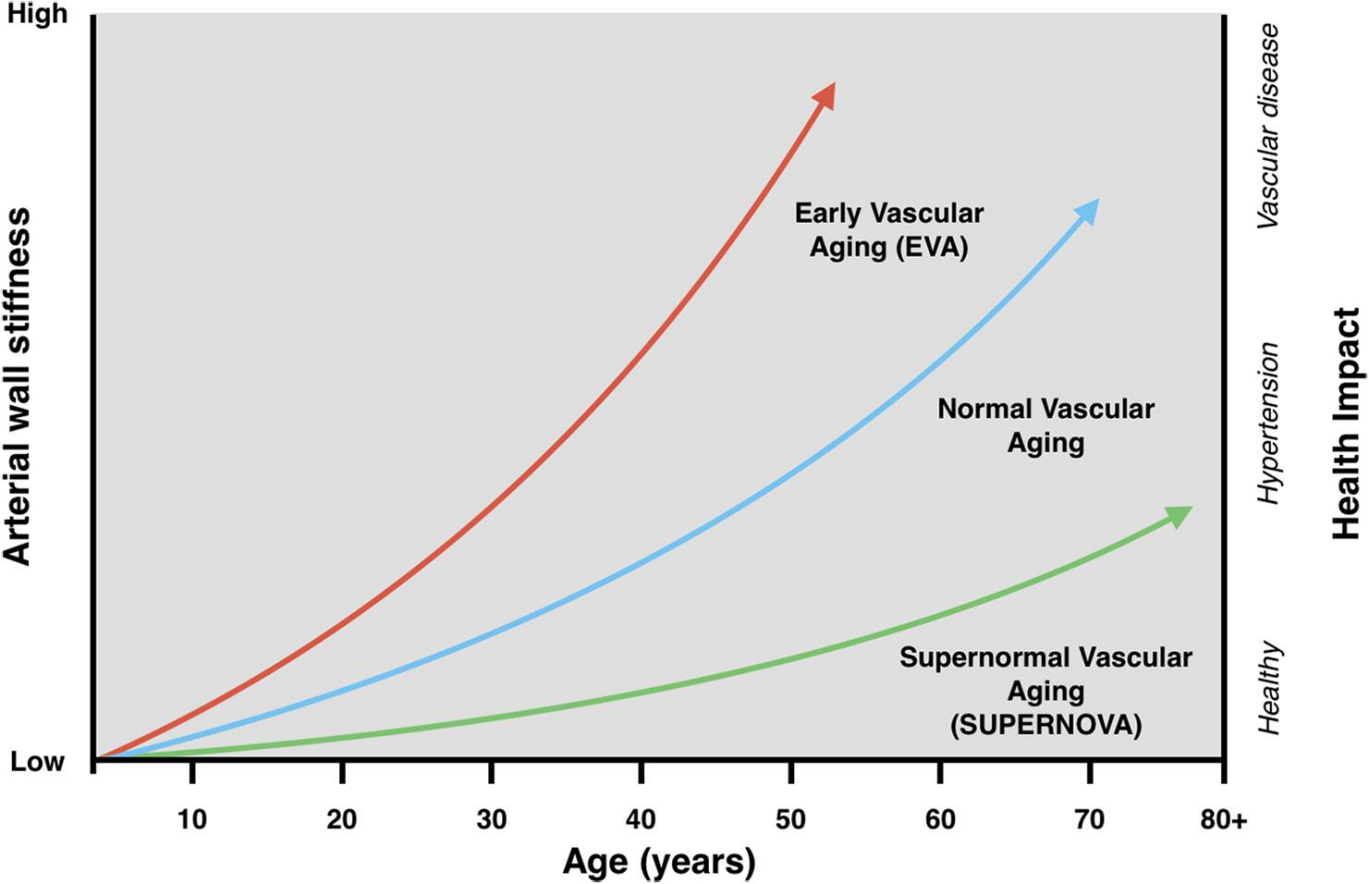
Conceptual relationship between biological and chronological vascular aging. Arterial stiffening progresses with age in all individuals, but those with Early Vascular Aging (EVA) exhibit accelerated stiffening, reaching pathological thresholds earlier in life. In contrast, individuals with supernormal vascular aging (SUPER-NOVA) demonstrate preserved stiffness and reduced cardiovascular risk.

Hypertension is a chronic elevation in blood pressure that gradually alters the structure and function of the vascular system. Constant mechanical stress on arterial walls promotes collagen buildup, elastin breakdown, and endothelial dysfunction, which together reduce arterial elasticity and increase stiffness (1, 4, 15, 16). Declining nitric oxide availability and excess oxidative stress further impair vasomotor control, amplifying inflammation and vascular injury (15, 16). These changes raise PWV and systolic pressure, increasing cardiac workload and accelerating vascular aging (4, 6). The interaction between sustained pressure, oxidative imbalance, and inflammation creates a self-perpetuating cycle that drives progressive arterial damage (see Figure 2).

Neurohormonal and inflammatory signaling intensify these effects. Activation of the renin–angiotensin–aldosterone system generates reactive oxygen species, stimulates smooth-muscle growth, and promotes fibrosis within the vessel wall (17–19). As stiffness and wave reflection increase, central pressure and afterload rise, linking mechanical strain to molecular injury (9). Over time, large and small arteries lose their normal compliance and regulatory capacity, producing early vascular aging characterized by reduced elasticity and impaired tissue perfusion.

At the cellular level, hypertension accelerates endothelial and smooth-muscle cell senescence. Endothelial dysfunction blunts vasodilation, while smooth-muscle remodeling and fibrosis limit contractility and vessel tone (8, 20, 21). Mitochondrial redox imbalance contributes to these alterations by reducing energy efficiency and repair capacity (22). The resulting microvascular rarefaction increases peripheral resistance and end-organ vulnerability. Noninvasive assessments such as carotid–femoral and brachial–ankle pulse-wave velocity provide practical measures of arterial stiffness and vascular age in hypertensive patients (23). Together, these mechanisms illustrate how hypertension not only damages the vasculature but also accelerates the biological aging process of the cardiovascular system.

### Coronary artery disease

Coronary artery disease represents one of the most well-characterized models of accelerated vascular aging, and it highlights how chronic inflammation and oxidative damage accelerate the process. The process begins when low-density lipoprotein (LDL) enters the arterial intima and undergoes oxidative modification, forming oxidized LDL (oxLDL). This triggers local inflammation, attracting macrophages and T cells, which engulf the lipids to form foam cells. Over time, this leads to necrosis, fibrous cap remodeling, and plaque formation, all of which damage the endothelium and stiffen the vessel wall, contributing to an EVA phenotype (21, 22, 24, 25) (see Figure 2). These changes are not limited to large coronary arteries; microvascular dysfunction frequently develops alongside plaques, reducing coronary flow and impairing tissue perfusion even in the absence of severe stenosis (11, 25).

Endothelial dysfunction in CAD acts as both a driver and a consequence of disease progression. A dysfunctional endothelium becomes more permeable, proadhesive, and prothrombotic, allowing further lipid entry and immune cell adhesion. As plaques develop, they disrupt endothelial signaling and impair the microcirculatory system, which increases local inflammation and vascular injury (20, 21).

Patients with CAD often demonstrate features of accelerated vascular aging, including increased arterial stiffness, elevated pulse wave velocity, and endothelial dysfunction appearing earlier than expected for their chronological age (2, 10, 11). In the perioperative context, these vascular changes increase susceptibility to hemodynamic instability, impaired tissue perfusion, and delayed wound healing (25).

### Heart failure with preserved ejection fraction

In HFpEF, macrovascular stiffness and endothelial dysfunction are central pathophysiologic contributors increasing arterial load and impairing perfusion. This stresses the myocardium decreasing cell longevity leading to local cellular fibrosis and impairing cardiac function over time. Larson et al. highlight that aging myocardium has a lower mitochondrial reserve and responds more poorly to hemodynamic shifts that the body may undergo. This decrease in vascular responsiveness closely mirrors what we see systemically in individuals with EVA (14).

In the IDENTIFY-HF study patients with HFpEF were found to have significantly higher PWV when compared against age matched controls. This finding seems to suggest that vascular stiffness occurs more rapidly in individuals with HFpEF and that these vascular changes positively correlate with an increasing number of comorbidities (12). This elevated afterload represented by a higher PWV contributes to poor ventricular-arterial coupling, restricting diastolic filling and putting further stress on the myocardium. As a result, we see higher systolic pressures systemically and worse overall microvascular perfusion and nutrient supply, a hallmark of EVA 26. By looking at HFpEF as not simply an issue of impaired relaxation but as a vascular syndrome where increased stiffness exacerbates underlying endothelial damage and inflammatory pathways, we can see how it closely links to accelerated biologic aging (12).

Obokata et al. found that patients with HFpEF showed reduced cardiac reserve during times of high demand exemplified through exercise (13). This is in part due to increased cardiomyocyte turnover due to episodes of low-grade ischemia. This increase in cell turnover promotes fibrotic changes and limits the heart's ability to increase output during operative or inflammatory stressors. Ischemic injury over time leads to systemic inflammation and oxidative radical formation, which are key aspects of EVA. These mechanisms eventually lead to vascular stiffening beyond the myocardium. The connection between systemic consequences and reduced cardiac reserve seen in HFpEF demonstrates that this condition is a consequence and amplifier of the vascular aging phenotype. This is particularly true for patients with other comorbidities such as hypertension or coronary artery disease (13).

### Clinically assessing vascular aging and operative implications

Hamczyk et al. demonstrated that biological aging markers are more closely tied to functional decline and the pathophysiological response to stress than an individual's chronological age alone. This suggests that vascular age may provide a better picture of surgical and anesthetic candidacy preoperatively (27). During surgical stresses the microcirculation can be heavily impacted, factors such as vascular stiffening and endothelial dysfunction worsen the microcirculations adaptability (22). Li et al. highlights that impaired vascular compliance is associated with compromised microcirculatory flow, delays in wound healing and poor tissue oxygenation all of which are more common in those with early vascular aging (28). The kidneys are of particular concern in individuals suffering from early vascular aging. Arterial stiffness transmits excessive pulsatile energy into renal vasculature increasing susceptibility to acute kidney injuries during large shifts in hemodynamics (29).

Arterial stiffness can be assessed through cfPWV which is considered a noninvasive standard. cfPWV can be measured through the transit time of an arterial pressure waveform between the carotid and femoral arteries using applanation tonometry or through oscillometric sensors that have been synchronized with electrocardiography. It should be noted that these systems require FDA approved equipment and training, potentially limiting immediate implementation, however patient burden is low, and testing can be completed fairly quickly. It is important that perioperative anesthetic management be augmented with these difficulties in mind. While it would be impractical to cancel surgeries due to the findings of increased vascular age clinicians can focus on keeping blood pressure targets narrower, slower administration of vasoactive medications and limiting unnecessary crystalloids as ways to mitigate potential harm (30).

In addition to direct measures of arterial stiffness, readily available clinical and biochemical markers provide integrative insight into vascular aging. Electrocardiographic abnormalities, particularly left ventricular hypertrophy, reflect chronic pressure overload and cumulative vascular stress and are independently associated with increased all-cause mortality, even among individuals classified as low risk by traditional criteria, supporting their role as markers of advanced vascular aging (31). Elevated high-sensitivity C-reactive protein levels, particularly values exceeding 2 mg/L, are associated with adverse metabolic profiles and age-related vascular changes in otherwise healthy populations, supporting hs-CRP as a biomarker of inflammatory vascular aging (32). Functional assessments, such as the six-minute walk test, may further refine vascular age estimation by identifying impaired cardiovascular and vascular reserve not apparent at rest, particularly in patients with preserved ejection fraction heart failure or multiple vascular comorbidities.

## Discussion

The concept of vascular age offers a physiologic framework partly explaining why individuals who have comorbidities such as hypertension, coronary artery disease and heart failure may seem less adaptable perioperatively than their chronologic age would suggest. Early vascular aging arises from the combination of three mechanisms, endothelial dysfunction, oxidative stress and chronic inflammation (6, 15, 16). These shared mechanisms work in tandem to accelerate an individual's vascular age and arterial stiffness beyond what would be expected for their chronologic age (1). This review highlights how HTN, CAD and HFpEF lead to EVA through similar but independent physiologic mechanisms.

Hypertension models how chronic pressure overload induces structural remodeling leading to, collagen deposition, elastin fragmentation, involvement of the RAAS system and angiotensin II mediated oxidative stress (17). These changes contribute to macrovascular and microvascular dysfunction leading to increased PWV, impaired cardiac reserve and increased susceptibility to complications resulting from sudden hemodynamic changes (8, 9).

Coronary artery disease triggers inflammatory cell recruitment through the modification of LDL, forming foam cells and promoting plaque development (21, 24). Dysfunction of the endothelial cell layer is a cause and consequence of this inflammatory pathway. The EVA phenotype impacts both the microvascular system and plays a role in macrovascular stiffening causing a decreased coronary flow reserve worsening dysfunction during stress (10, 11, 25).

Heart failure illustrates how vascular aging through systemic arterial stiffening can lead to a rise in afterload increasing myocardial oxygen and damage. Microvascular issues further impair nutrient delivery and contribute to diastolic dysfunction. These vascular abnormalities contribute to progression of heart failure and also signify early vascular aging (14, 27).

## Conclusion

Across these disease states vascular age seems to more accurately capture an individual's functional reserve, tissue perfusion capacity and perioperative risk than their chronological age would alone. Advances in imaging and biomarkers now make vascular age determination more feasible in clinical settings and likely have prognostic value independent of traditional risk factors. Future research should be aimed toward refining vascular age scoring systems, incorporating these scoring systems into perioperative decision-making algorithms and potentially integrating these scoring systems into electronic medical records for ease of use. By looking at an individual's vascular age instead of chronologic age clinicians should be able to more accurately stratify surgical risk, tailor intraoperative management and identify potential complications earlier. Through this shift clinicians will improve clinical outcomes for patients whose vascular age differs significantly from their chronological age.
